# Unmasking the Uncommon Arc of Buhler Aneurysm: A Report of Two Cases

**DOI:** 10.7759/cureus.89351

**Published:** 2025-08-04

**Authors:** Reshmi Elangovan, Rajoo Ramachandran, Harini Gnanavel

**Affiliations:** 1 Radiology and Imaging Sciences, Sri Ramachandra Institute of Higher Education and Research, Chennai, IND

**Keywords:** arc of buhler aneurysm, celiac artery stenosis, collateral circulation, ct angiography, endovascular embolization, visceral artery aneurysm

## Abstract

The Arc of Buhler (AOB) is a rare embryological vascular remnant that forms a persistent arterial connection between the superior mesenteric artery and the celiac artery. Although typically asymptomatic, it assumes clinical importance in the presence of celiac artery stenosis by serving as a key collateral route. Aneurysms arising from the AOB are uncommon but carry a significant risk of rupture. This report presents two cases of AOB aneurysms identified in different clinical scenarios. The first case involved a 40-year-old woman with chronic epigastric discomfort, in whom a saccular aneurysm of the AOB was detected alongside celiac artery narrowing. The second case was of a 62-year-old man evaluated for abdominal trauma, when an incidental aneurysm of the AOB was observed on contrast-enhanced CT. In both instances, imaging revealed retropancreatic aneurysmal dilatation and stenosis of the celiac axis, suggesting altered hemodynamics with increased collateral flow as a possible cause. Key diagnostic tools include contrast-enhanced CT, CT angiography, and digital subtraction angiography. Endovascular embolization remains the preferred treatment approach, with surgical intervention reserved for select situations. Recognizing this rare vascular anomaly is essential to prevent intraoperative complications and ensure proper management. Despite its rarity, an AOB aneurysm should be considered in cases of visceral aneurysm, particularly with concurrent celiac artery stenosis.

## Introduction

The Arc of Buhler (AOB) is a rare vascular remnant of an embryonic anastomosis between the superior mesenteric artery (SMA) and celiac artery (CA). First identified by Buhler in 1904, this arterial connection is generally asymptomatic and often incidentally discovered on imaging or surgery [1]. However, in case of CA occlusion or stenosis, the AOB can be hemodynamically significant as an important collateral pathway [2,3]. This can expose the vessel to increased hemodynamic stress and predispose it to aneurysmal degeneration [4].

Aneurysm of the AOB is extremely rare and dangerous due to the risk of rupture [5,6]. It typically presents incidentally on imaging for unrelated symptomatology in the abdomen or trauma, but also may present with catastrophic hemorrhage or nonspecific abdominal pain [6,7]. Its clinical relevance has been underscored recently in the literature because of the progress with cross-sectional imaging modalities like contrast-enhanced computed tomography (CECT) and computed tomography angiography (CTA), which make visceral arterial anomalies better visualized [8]. Owing to its potential for severe complications and the rarity of the disease, heightened clinical suspicion and rapid diagnosis are critical. This report describes two such instances of AOB aneurysms that were found under different clinical situations, bringing to focus the diagnostic significance and therapeutic difficulties posed by this vascular anomaly.

## Case presentation

Case 1

A 40-year-old woman presented with three months of non-radiating, dull epigastric pain. She had no other gastrointestinal symptoms, previous abdominal operations, or relevant medical comorbidities. Physical examination was normal, and her initial laboratory evaluation revealed normal complete blood counts and liver function tests. But serum amylase and lipase levels were increased, raising the suspicion of a pancreatic or peripancreatic cause (Table 1).

**Table 1:** Laboratory investigations A: G ratio: albumin-to-globulin ratio

Tests	Patient Value	Unit	Reference Range
TOTAL BLOOD COUNTS			
White blood cells	9.5	(x 10 ^9^/L)	4.5-11
Haemoglobin	11.5	g/dl	11-13
Platelets	245	(x 10 ^9^/L)	150-400
BIOCHEMICAL TESTS			
Serum amylase	222	U/L	22-80
Serum Lipase	356	U/L	<67
LIVER FUNCTION TESTS			
Aspartate aminotransferase (AST)	34	IU/L	<35
Alanine aminotransferase (ALT)	32	IU/L	<35
Alkaline phosphatase	58	IU/L	30-120
Total protein	5.4	g/dl	6.6-8.3
Albumin	3.1	g/dl	3.5-5.2
Globulin	2.3	g/dl	2 – 3.5
A:G ratio	1.3	-	-
Total Bilirubin	0.84	mg/dL	0.3-1.2
Direct Bilirubin	0.23	mg/dL	0.0-0.3
Indirect Bilirubin	0.61	mg/dL	0.2-0.8

**Figure 1:**
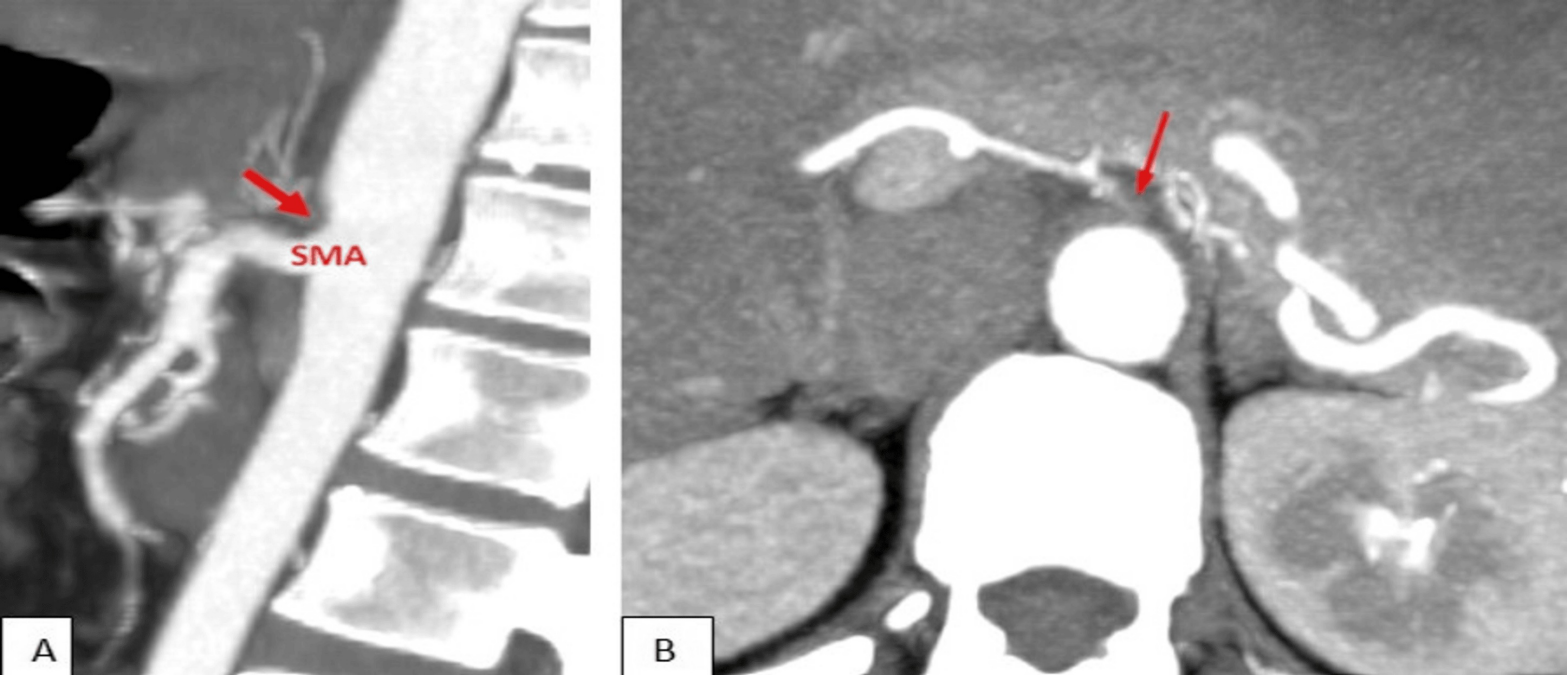
CECT abdomen, sagittal (A) and axial (B) views, of a 40-year-old woman with pain in abdomen (Case 1) Maximum intensity projection (MIP) images of the arterial phase show complete non-visualization of the celiac trunk with several small arterial collaterals in the region where the celiac artery distribution was expected CECT: contrast-enhanced computed tomography; SMA: superior mesenteric artery

A CECT abdomen was done, which showed non-visualization of the celiac trunk with several small arterial collaterals in the region where the celiac artery distribution was expected (Figure 1).

**Figure 2:**
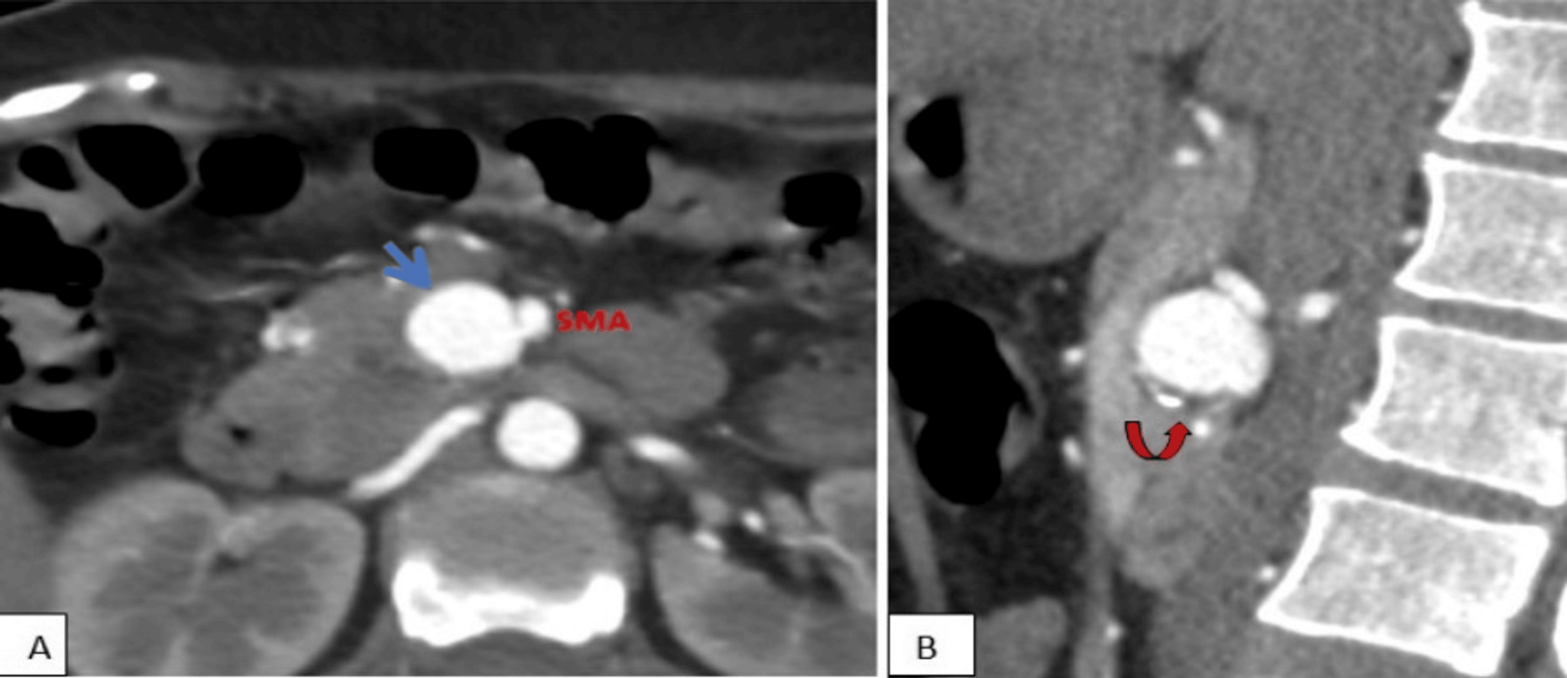
CECT abdomen of a 40-year-old women with pain in abdomen diagnosed with AOB aneurysm (Case 1) (A) Axial section in the arterial phase showed the saccular AOB aneurysm (blue arrow )in close relation with the SMA at the peripancreatic region; (B) Reformatted sagittal view in the arterial phase shows eccentric intramural thrombus and focal wall calcifications of the AOB aneurysm (curved arrow). CECT: contrast-enhanced computed tomography; AOB: Arc of Buhler; SMA: superior mesenteric artery

**Figure 3:**
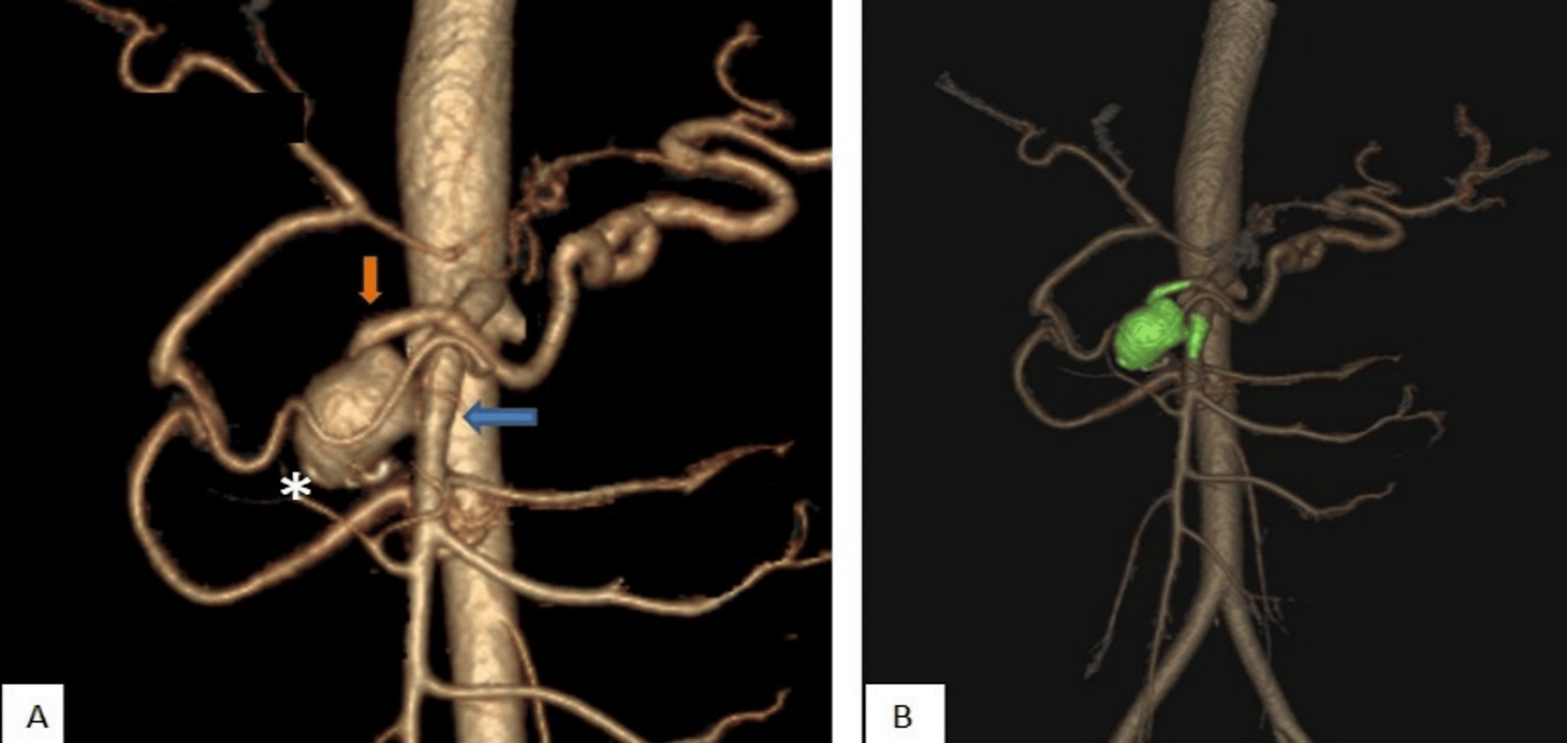
CECT volume-rendered imaging (Case 1) (A) AOB aneurysm (asterisk) is seen in close relation with the SMA (blue arrow) with a collateral branch (orange arrow) arising from its postero-lateral aspect, seen to continue as the splenic artery (a branch of celiac artery); (B) Arch of Buhler is seen between the SMA and the suspected celiac bed with an aneurysm originating from this abnormal artery CECT: contrast-enhanced computed tomography; AOB: Arc of Buhler; SMA: superior mesenteric artery

A saccular aneurysm measuring 1.9 × 2.4 × 1.8 cm was seen in the peripancreatic region, in close relation with the SMA (Figure 2A). The aneurysm had eccentric intramural thrombus and focal wall calcifications indicative of a chronic process (Figure 2B). Further, a collateral branch arising from its postero-lateral aspect is seen to continue as the splenic artery (a branch of CA)( Figure 3A). These characteristics established the diagnosis of an AOB aneurysm, clearly outlining a persistent anastomotic vessel between the SMA and the suspected celiac bed, with the aneurysmal dilatation originating from this abnormal artery (Figure 3B).

The patient was referred to the department of vascular surgery for further management and underwent open aneurysmal repair and revascularization surgery for CA stenosis. Postoperatively, the patient improved symptomatically and was followed up regularly for one year.

Case 2

**Figure 4:**
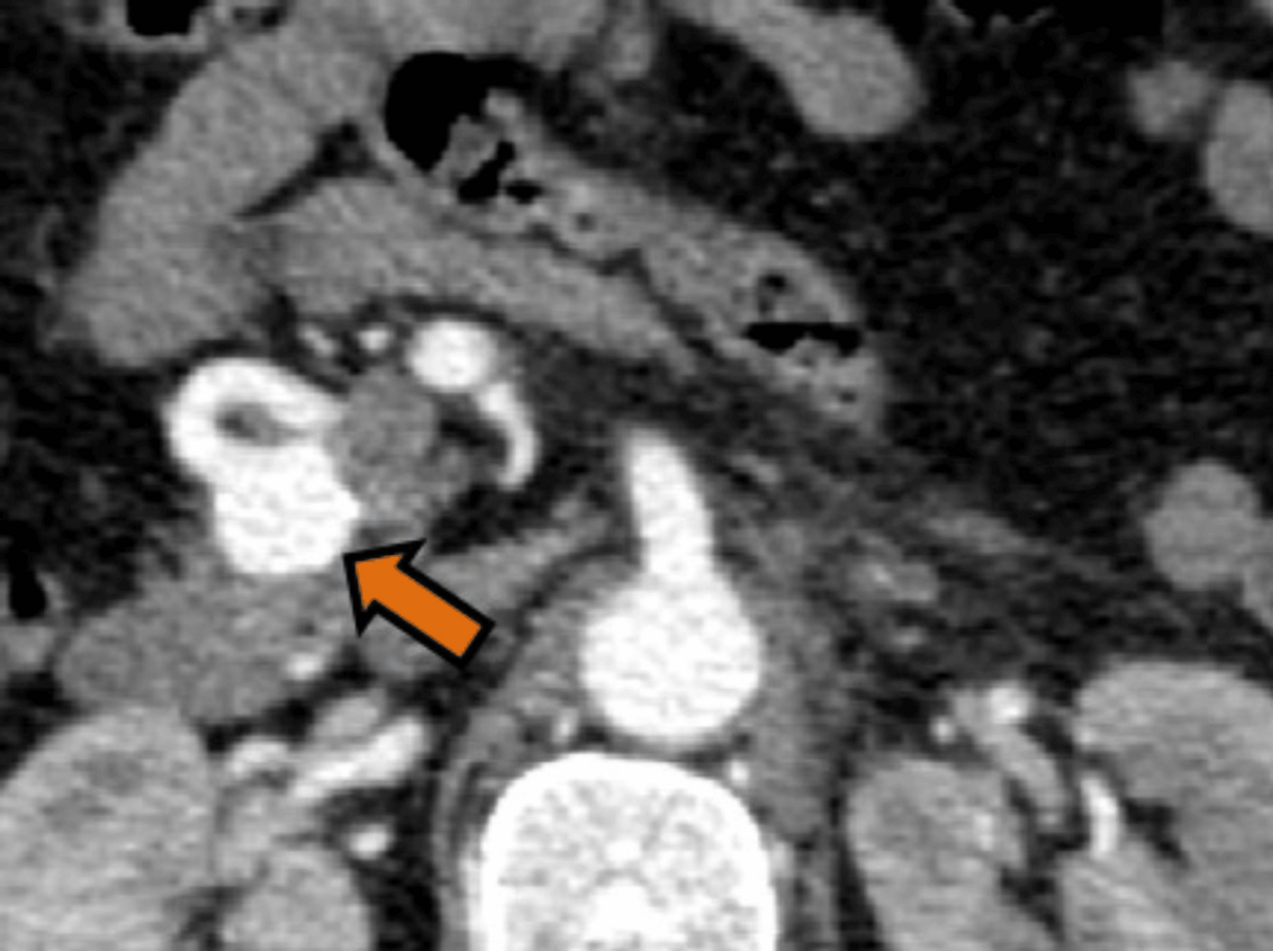
CECT of a 62-year-old trauma victim with incidentally diagnosed AOB aneurysm (Case 2) Axial section of arterial phase shows an aneurysm with few eccentric wall calcifications noted in close proximity along the course of SMA (orange arrow) in the head of the pancreas region CECT: contrast-enhanced computed tomography; SMA: superior mesenteric artery

A 62-year-old man presented to the emergency department after a high-impact road traffic accident. He was hemodynamically stable but had undergone abdominal imaging to exclude intra-abdominal injuries. CECT was negative for any solid organ injury, but incidentally detected an aneurysm measuring 1.9 × 1.7 cm with eccentric wall calcifications seen in close proximity to the path of the SMA; adjacent to the head of the pancreas (Figure 4).

**Figure 5:**
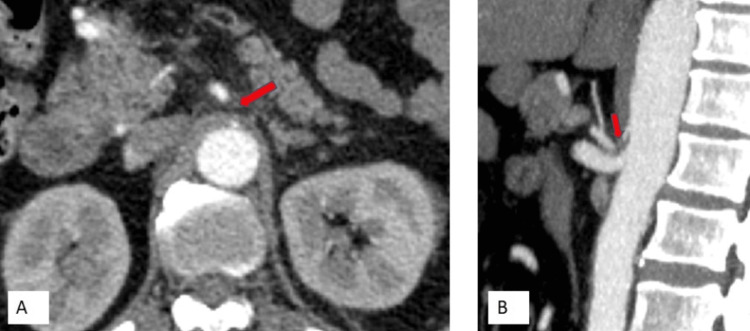
CECT of a 62-year-old trauma victim with incidentally diagnosed AOB aneurysm (Case 2) Axial (A) and sagittal (B) sections showing showing short segment complete stenosis of the celiac trunk near its origin CECT: contrast-enhanced computed tomography; AOB: Arc of Buhler

**Figure 6:**
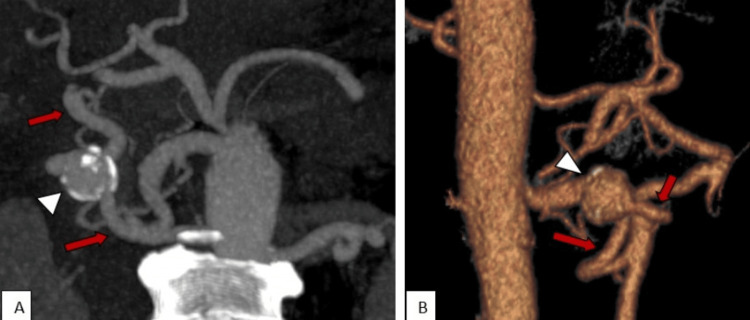
CECT of a 62-year-old trauma victim with incidentally diagnosed AOB aneurysm (Case 2) Maximum intensity projection (MIP) image (A) and volume-rendered imaging (B) in arterial phase showing the AOB between the SMA and the celiac territory (red arrows) with an aneurysm (white arrow head) originating from this collateral vessel. CECT: contrast-enhanced computed tomography; AOB: Arc of Buhler; SMA: superior mesenteric artery

The arterial phase was carefully studied to further assess the vascular anatomy. This validated short-segment celiac artery stenosis (Figure 5) and a well-formed collateral vessel between the SMA and the celiac territory, in keeping with an AOB that persisted; and the above-mentioned aneurysm arose from this abnormal vessel (Figure 6).

The vascular results were scrutinized by a multidisciplinary panel of interventional radiology, vascular surgery, and gastroenterology. Due to the patient's stable status and the incidental nature of the discovery, he was a candidate for elective endovascular repair of the aneurysm. The postoperative period was uneventful, and hence was discharged and regularly followed up for six months.

## Discussion

The AOB is an uncommon embryological remnant artery that is a direct anastomosis between the SMA and the celiac trunk, circumventing the normal pancreaticoduodenal arcade [1,8]. During embryogenesis, a few ventral splanchnic arteries emerge between the aorta and the gastrointestinal tract. These would usually regress, leaving us with the definitive celiac trunk and SMA. Persistence of some of these segments leads to anatomical variants such as the AOB [2]. Though it is infrequent, found in 1-4% of imaging studies [8], its identification is important in many clinical scenarios.

In the two cases presented in this report, AOB aneurysms were both linked to celiac axis stenosis, validating the theory that enhanced hemodynamic stress from compensatory flow through this collateral artery could be a factor in aneurysm development [4,3]. Visceral artery aneurysms in collateral arteries, especially with associated upstream stenosis, are all implicated in the pathogenesis by chronic shear stress, turbulent flow, and degeneration of the arterial wall [4,5].

The clinical presentation of AOB aneurysms is very varied. Some patients, such as the initial case, can present with abdominal pain that is chronic in duration, whereas others, such as in the second case, can have totally asymptomatic aneurysms found incidentally [7,9]. In the minority of cases, patients can present with acute hemorrhage or with complications such as biliary obstruction, pancreatitis, or sometimes with life-threatening retroperitoneal hemorrhage secondary to rupture [6,7]. Because of this broad clinical spectrum, a great index of suspicion is required when assessing peripancreatic vascular lesions.

Imaging is the key to the diagnosis of AOB aneurysms. CECT and CT angiography are the modalities of choice, with outstanding spatial resolution and the capacity to map the vascular anatomy in detail [8,3]. Digital subtraction angiography is still considered the gold standard for vascular assessment, but it is generally reserved for pre-interventional planning or if endovascular therapy is under consideration [5].

Endovascular repair has become the treatment of choice in stable patients with AOB aneurysms [5]. Procedural techniques involve coil embolization, stent graft insertion, or liquid embolic agent use, based on aneurysm morphology and vascular anatomy. When endovascular access is challenging or contraindicated, open surgical repair can be required [10]. Treatment selection must be tailored to individual patient factors, aneurysm diameter, rupture risk, and comorbid conditions.

The two cases presented illustrate the range of clinical presentations and underscore the need to include vascular anomalies in the differential diagnosis of abdominal pain or their incidental discovery. Furthermore, the co-occurrence with stenosis of the CA in both patients underscores the necessity of checking for narrowing of the proximal vessel when an AOB aneurysm is discovered [4,3].

## Conclusions

AOB aneurysms, although uncommon, are of major clinical significance, particularly when involving CA stenosis. Their recognition has grown with the introduction of high-resolution cross-sectional imaging, allowing precise anatomical delineation and prompt intervention. The cases presented in this report show the wide range of clinical settings in which AOB aneurysms may be found and highlight imaging's role in diagnosis and treatment. Recognition of this anatomic variation is vital in interventional procedure planning, avoiding complications, and maximizing patient outcomes.
